# A practical guide to the implementation of AI in orthopaedic research, Part 6: How to evaluate the performance of AI research?

**DOI:** 10.1002/jeo2.12039

**Published:** 2024-05-31

**Authors:** Felix C. Oettl, Ayoosh Pareek, Philipp W. Winkler, Bálint Zsidai, James A. Pruneski, Eric Hamrin Senorski, Sebastian Kopf, Christophe Ley, Elmar Herbst, Jacob F. Oeding, Alberto Grassi, Michael T. Hirschmann, Volker Musahl, Kristian Samuelsson, Thomas Tischer, Robert Feldt, ESSKA Artificial Intelligence Working Group

**Affiliations:** ^1^ Hospital for Special Surgery New York New York USA; ^2^ Schulthess Klinik Zurich Switzerland; ^3^ Sports Medicine and Shoulder Institute, Hospital for Special Surgery New York New York USA; ^4^ Department for Orthopaedics and Traumatology, Kepler University Hospital GmbH Johannes Kepler University Linz Linz Austria; ^5^ Department of Orthopaedics, Institute of Clinical Sciences, Sahlgrenska Academy University of Gothenburg Gothenburg Sweden; ^6^ Sahlgrenska Sports Medicine Center Göteborg Sweden; ^7^ Department of Orthopaedic Surgery Tripler Army Medical Center Honolulu Hawaii USA; ^8^ Department of Health and Rehabilitation, Institute of Neuroscience and Physiology, Sahlgrenska Academy University of Gothenburg Gothenburg Sweden; ^9^ Center of Orthopaedics and Traumatology, University Hospital Brandenburg an der Havel, Brandenburg Medical School Theodor Fontane Germany; ^10^ Department of Mathematics University of Luxembourg Esch‐sur‐Alzette Luxembourg; ^11^ Department of Trauma, Hand and Reconstructive Surgery University Hospital Muenster Muenster Germany; ^12^ Mayo Clinic Alix School of Medicine, Mayo Clinic Rochester Minnesota USA; ^13^ IIa Clinica Ortopedica e Traumatologica, IRCCS Istituto Ortopedico Rizzoli Bologna Italy; ^14^ Department of Orthopaedic Surgery and Traumatology Kantonsspital Baselland Bruderholz Switzerland; ^15^ University of Basel Basel Switzerland; ^16^ Department of Orthopaedic Surgery, UPMC Freddie Fu Sports Medicine Center University of Pittsburgh Pittsburgh Pennsylvania USA; ^17^ Department of Orthopaedics Sahlgrenska University Hospital Mölndal Sweden; ^18^ Department of Orthopaedic Surgery Universitymedicine Rostock Rostock Germany; ^19^ Department of Orthopaedic and Trauma Surgery Malteser Waldkrankenhaus Erlangen Erlangen Germany; ^20^ Department of Computer Science and Engineering Chalmers University of Technology Gothenburg Sweden

**Keywords:** AI, digitalization, healthcare, ML, performance metrics

## Abstract

Artificial intelligence's (AI) accelerating progress demands rigorous evaluation standards to ensure safe, effective integration into healthcare's high‐stakes decisions. As AI increasingly enables prediction, analysis and judgement capabilities relevant to medicine, proper evaluation and interpretation are indispensable. Erroneous AI could endanger patients; thus, developing, validating and deploying medical AI demands adhering to strict, transparent standards centred on safety, ethics and responsible oversight. Core considerations include assessing performance on diverse real‐world data, collaborating with domain experts, confirming model reliability and limitations, and advancing interpretability. Thoughtful selection of evaluation metrics suited to the clinical context along with testing on diverse data sets representing different populations improves generalisability. Partnering software engineers, data scientists and medical practitioners ground assessment in real needs. Journals must uphold reporting standards matching AI's societal impacts. With rigorous, holistic evaluation frameworks, AI can progress towards expanding healthcare access and quality.

**Level of Evidence:**

Level V.

**Abbreviations**
AI: artificial intelligence
AUC‐ROC: area under the curve‐receiver operator curve
BLEU: bilingual evaluation understudy
DVT: deep venous thrombosis
FN: false negative
FP: false positive
LLM: large language models
MAE: mean absolute error
MAP: mean average precision
MAPE: mean absolute percentage error
MCC: Matthews Correlation Coefficient
ML: machine learning
NDCG: normalised discounted cumulative gain
RMSE: root‐mean squared error
TN: true negative
TP: true positive
TRIPOD: transparent reporting of a multivariable prediction model for individual prognosis or diagnosis

## INTRODUCTION

*All models are wrong, but some are useful*.

George Box [4]

The rapid development of artificial intelligence (AI) has led to systems capable of predicting outcomes, analysing and reporting on data produced by imaging procedures, as well as generating creative works such as generated music, graphics or even art. However, as these powerful technologies advance, it is essential that their outputs are properly interpreted and evaluated, in particular, for applications in medicine. While high predictive capacity is desirable, values nearing or reaching 100% should be examined closely to rule data leakage or other methodological concerns. Very small sample sizes can make models prone to overfitting. Respective studies warrant careful scrutiny regarding their evaluation methodology and the appropriateness of any claims.

Machine learning (ML) models have shown promising predictive abilities for select tasks in medicine, such as screening skin lesions for cancer risk or predicting protein folding structures [16, 43]. However, ML models' ability to make reliable clinical judgements across all domains of medicine remains limited at this time. While ML algorithms can find patterns and correlations in data, correlations alone are not sufficient to justify clinical actions for specific patient care. To make credible clinical judgements, or even to support the decisions of medical staff, AI needs access to extensive medical data and an understanding of diagnostic methods, treatment options and surgical techniques grounded in clinical expertise. For example, an ML algorithm may discover gene mutations associated with increased disease risk, but qualified medical professionals must validate and interpret these findings to determine appropriate next steps for each patient and put these findings into context with what is already known about the pathophysiology and nature of the disease. More research is needed to develop AI systems with the reasoning capabilities necessary for sound clinical decision‐making [8, 20, 21, 25, 44, 51, 52, 53, 55, 56].

To enable proper evaluation, researchers should report on the ML model's training process, data sources, modelling capabilities and performance [9, 31]. Comparisons to ground truth benchmarks, measured error rates and comparisons with human experts can contextualise the AI's performance. Additionally, AI outputs should be critically examined through peer review, replication studies and real‐world, clinical testing before being integrated into standard practices. This process is known as external validation, where models are tested on data sets from different patient populations, geographic locations or hospitals to ensure that no bias was introduced during the internal training process. With thoughtful evaluation methods, AI can augment human performance and ameliorate patient outcomes. Standards for reporting and interpretation of AI and ML model performance can help in this, but it is important to acknowledge that evaluations may need to be reassessed if a model is used over time. The underlying population of patients, and thus the data, might drift, potentially rendering the models obsolete.

## ML MODELS AND METRICS

While generative AI (ChatGPT [OpenAI Inc.] probably being the most well‐known in 2024) is becoming increasingly important in all fields of science, the three main applications utilising AI in healthcare remain classification, regression and clustering [11, 35, 47]. Other ML algorithms are often permutations and combinations of these underlying models. Commonly proposed algorithms in medicine that define themselves ‘Ranking’ or ‘Forecasting’ are permutations and/or combinations of Classification, Regression and Clustering models.

### Classification

A common use of AI is to classify (e.g., X‐rays) into discrete categories or labels [36]. For example, an image classifier may categorise X‐rays as normal or abnormal [34]. Classification models output a predicted class, possibly along with a probability score reflecting the model's confidence. Key evaluation metrics are confusion matrices from which accuracy, precision, recall, specificity and F1‐scores, as well as area under the curve–receiver operator curve (AUC‐ROC) can be calculated [36]. Arguments have been made that if only a single metric is to be used, then the Matthews Correlation Coefficient (MCC) has many benefits since it summarises all the other basic rates (sensitivity, specificity, precision and negative predictive value) while AUC‐ROC does not [6]. However, high scores on internal validation (commonly referred to as test set) do not necessarily mean that AI will generalise to real‐world use [15, 54]. When assessing classification models, it is vital to critically evaluate how accurately the distribution of ground truth labels represents the true underlying prevalence across classes. Real‐world data sets often exemplify class imbalance, where the positive disease cases comprise a disproportionately smaller fraction compared to the negative cohort. For example, in a prediction model for deep venous thrombosis (DVT) after surgery, only a fraction of the patient population will present with DVT, this is called an imbalanced data set.

### Regression

Regression models predict continuous numeric values instead of discrete classes, such as patient length of stay based on clinical data [36]. Evaluation focuses on deviation from true values, using metrics like percentiles of errors, mean absolute error (MAE), mean absolute percentage error (MAPE) and root‐mean‐squared error (RMSE) [24, 36, 38]. However, solely chasing better numeric scores can overlook whether outputs are clinically meaningful [29].

### Clustering

Evaluating the performance of unsupervised clustering algorithms requires metrics that quantify how well the clusters separate dissimilar observations and group similar ones [36]. A cluster refers to a set of observations that is more related to each other than to data points in other clusters. For example, a clustering algorithm may separate patients into distinct clusters based on symptoms and test results, with each cluster representing a potential undiscovered disease subtype. Two popular performance metrics are the Silhouette Coefficient and Dunn's Index [14, 42]. The Silhouette Coefficient measures how close each observation is to others in its cluster versus the next nearest cluster. It ranges from −1 (poor clustering) to +1 (dense, well‐separated clusters) [42]. The Dunn's Index computes the ratio of the minimal intercluster distance to the maximal cluster diameter. Here, distance refers to the chosen similarity metric used to compare data points during clustering. For medical data, this could be the Euclidean distance between feature vectors. Cluster diameter measures dispersion within a cluster by the greatest distance between any two members [14].

Larger intercluster separation gaps and more compact cluster sizes (lower diameter) produce higher Dunn's Index values, indicating better delineation of distinct groups. However, an inherent assumption is that the natural clusters in the data are dense and well‐separated [14].

In some medical contexts, underlying conditions may better manifest as overlapping, sparse or elongated clusters. For example, co‐morbidities could link symptoms of two diseases, preventing clean separation. In such cases, poor Dunn Index scores do not necessarily indicate ineffective clustering, but a mismatch between analysis assumptions and real‐world ambiguity. The clustering itself may still provide clinical utility. However, interpretation should account for complexity in the disease patterns defying assumptions. In these situations, different similarity metrics or clustering approaches optimised for interconnected data may be warranted [28].

### Recommendation and ranking

Recommendation and ranking systems suggest items likely of interest to a certain user or patient, such as research papers relevant to a surgeon's specialty, or a clinical study to a patient, based on their previous behaviour and known metrics.

Recommendation systems can be classified into two main categories: collaborative filtering and content‐based filtering [41]. Collaborative filtering algorithms recommend items based on the ratings or preferences of other users. For example, if you have rated a publication highly, a collaborative filtering algorithm might recommend other publications that have been rated highly by people who have similar interests as you. Content‐based filtering algorithms recommend items based on the features of the items themselves. For example, if you have read mainly arthroscopic literature, a content‐based filtering algorithm might recommend other arthroscopy‐related papers. Both approaches can be combined. In addition to collaborative filtering and content‐based filtering, recommendation systems can also use classification and regression algorithms. Classification algorithms are used to predict a categorical value, such as whether a user will like or dislike an item. Regression algorithms are used to predict a continuous value, such as the number of citations of an item.

In healthcare, such algorithms can be utilised to recommend patients clinical trials that they are more likely to participate in, online health resources that they are more likely to adhere to, and personalised lifestyle advice [46].

The performance of recommendation systems is commonly evaluated using metrics, such as precision, recall, mean average precision [3], normalised discounted cumulative gain (NDCG) and AUC‐ROC. The choice of performance metrics will depend on the specific application and the goals of the system. A key determinant is often how many recommended items are likely to be manually checked before a final selection is done.

### Forecasting and time series

AI forecasting leverages historical time series data to predict future values through ML regression techniques tailored to capture temporal patterns and trends [18, 27]. Common evaluation metrics for forecasting systems include MAE, RMSE and MAPE to quantify deviation from ground truth over the prediction horizon. While similar metrics are utilised for general regression tasks, the distinction lies in the explicit modelling of time‐based effects [45]. Judicious selection of appropriate skill metrics, testing on ample time series data encompassing variability, and reporting detailed performance across near‐term and longer‐range forecasts facilitates rigorous assessment of model accuracy and generalisability. Adoption of robust evaluation protocols tailored to the nature of forecasting problems enables standardised benchmarking and continued advancement of predictive technologies.

### Anomaly detection

Anomaly detection is an analytical task applied across various domains, notably in healthcare, cybersecurity and finance, to identify data instances that deviate from established norms [13, 49]. While its evaluation metrics exhibit some resemblances with classification, subtle distinctions emerge due to the unique data characteristics and primary objectives of anomaly detection. In the realm of anomaly detection, the foremost evaluation metrics include sensitivity, precision, recall, F1‐score and the AUC‐ROC. These metrics offer essential insights into the performance of anomaly detection algorithms. Anomaly detection frequently contends with imbalanced data sets wherein anomalies constitute a minority. Consequently, precision and recall assume heightened significance as anomalies necessitate meticulous scrutiny to minimise false alarms. This requires careful threshold definitions as this choice exerts a profound influence on the intricate precision‐recall trade‐offs inherent to anomaly detection.

In summation, anomaly detection is a specialised form of classification, notably within imbalanced data contexts, which necessitates a deliberate contemplation of precision and recall.

### Text generation

With the rise of ChatGPT (OpenAI Inc.), Claude 2 (Anthropic), Bard (Google) and other generative AI, text generation has already entered the healthcare system (e.g., chat bots) [10].

These large language models generate coherent text based on an initial text input that provides context and guides the direction of the output, known as a ‘prompt’. Outputs should be evaluated both automatically (grammar, coherence) and manually (factual correctness, creativity). An example of the former is the bilingual evaluation understudy (BLEU) which quantifies the degree to which a generated answer corresponds to a pre‐existing, expected one [37]. Kaarre et al. used expert orthopaedic surgeons as expert evaluators to judge responses from the GPT‐4 generative model [26]. Due to the nature of their output, these systems are notoriously hard to compare and contrast objectively. However, automatically calculated scores like BLEU have known limitations [5].

### Image/video generation

Generative deep‐learning models can create realistic images, audio and video (e.g., Dall‐E, Midjourney) [2]. The quality and fidelity of generated media can be measured via human evaluation, and similarity metrics such as Fréchet Inception Distance which uses deep neural network representations to quantify the statistical similarity of synthetically generated images compared to real images [22]. However, manipulation risks necessitate cautious deployment, and all output should undergo human review before being deployed.

In summary, AI outputs take a variety of numeric, textual and visual forms. Rigorously evaluating results for a given application requires selecting meaningful performance metrics, testing models on appropriate real‐world data, and considering changes in data over time. Interdisciplinary collaboration between technical and subject matter experts can help determine if AI outputs are reasonable, useful and generalisable.

## INTERPRETING AND EVALUATING OUTPUTS

Properly interpreting and evaluating AI outputs is crucial before applying models to real‐world tasks. Here, we further discuss aforementioned metrics and detail their interpretation.

### Evaluation metrics

Classification models output predicted labels and confidence scores. Metrics like accuracy, precision, recall, F1 and AUC‐ROC provide quantification, but have limitations, as no single metric fully captures performance [23, 36]. Nevertheless, in order to compare model performance, quantifiable performance metrics serve as an important tool. All these values rely on the underlying confusion matrix, summarising prediction of the model versus real‐world data. Four values are presented in a confusion matrix, similar in concept to methodology for assessing a new clinical diagnostic test:

**Table:** 

	Known positive	Known negative
Predicted positive	TP	FN
Predicted negative	FP	TN

- True positive (TP): Positive outcome correctly classified as positive.
- True negative (TN): Negative outcome correctly classified as negative.
- False positive (FP): Negative outcome incorrectly classified as positive.
- False negative (FN): Positive outcome incorrectly classified as negative.

$$\mathrm{Accuracy}=\frac{\left(\mathrm{TP}+\mathrm{TN}\right)}{\left(\mathrm{TP}+\mathrm{FP}+\mathrm{FN}+\mathrm{TN}\right)},$$

$$\mathrm{Precision}=\frac{\mathrm{TP}}{\left(\mathrm{TP}+\mathrm{FP}\right)},$$

$$\mathrm{Recall}=\frac{\mathrm{TP}}{\left(\mathrm{TP}+\mathrm{FN}\right)},$$

$$\mathrm{Sensitivity}=\frac{\mathrm{TP}}{\mathrm{TP}+\mathrm{FN}},$$

$$F1-\mathrm{score}=2\times \frac{\mathrm{precision}\times \mathrm{recall}}{\mathrm{precision}+\mathrm{recall}}.$$

Regression models' performance metrics derive from the value between the forecasted and actual variable of the test data [34]. Let $A_{t}$ and $F_{t}$ denote the actual and forecasted values of the test data point t, respectively. Then the MAPE is given by:

$$\mathrm{MAPE}=\frac{1}{n}\sum_{t=1}^{n}\left|\frac{A_{t}-F_{t}}{A_{t}}\right|,$$

while the RMSE corresponds to:

$$\mathrm{RMSE}=\sqrt{\frac{\sum_{t}{\left(A_{t}-F_{t}\right)}^{2}}{2}}.$$

The F1 − score, also known as the harmonic mean of precision and recall, is most useful for problems with imbalanced data set classes, as accuracy alone can be misleading if there is a majority negative class that is simple to predict [36, 39]. F1 handles class imbalance better as it incorporates precision and recall, considering true positives, false positives and false negatives. It ranges from 0 to 1, with 1 being a perfect prediction [39]. A drawback of the F1‐score is that it does not consider true negatives; however, it remains useful for healthcare, where minimising false positives and false negatives is critical [48].

**Figure 1:**
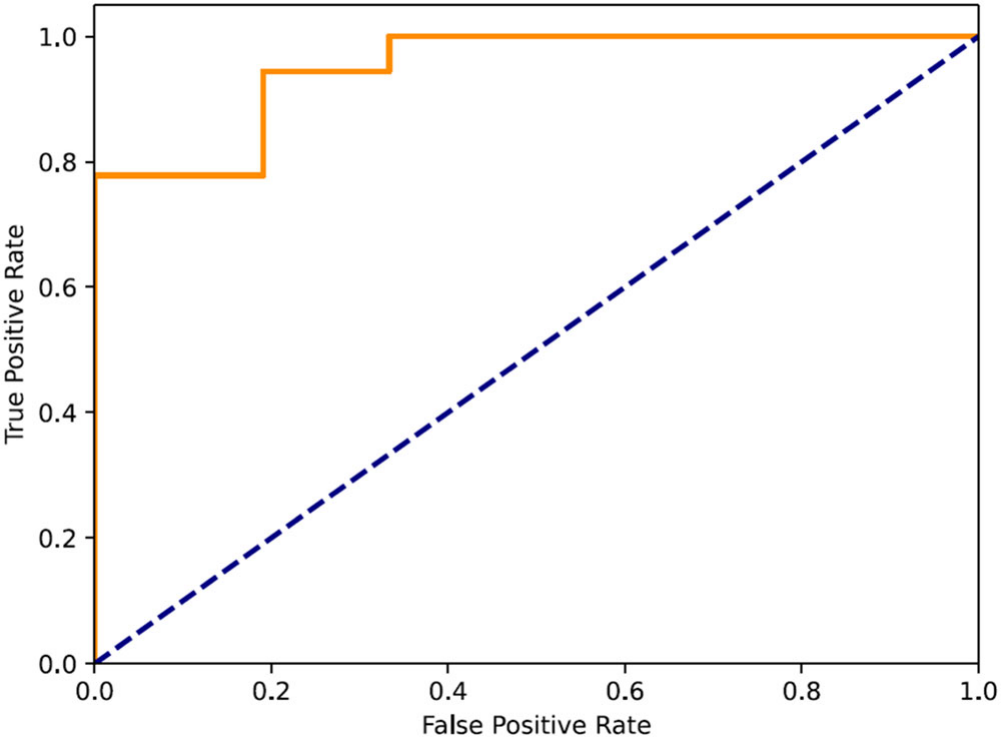
Area under the curve (AUC)–receiver operator curve (ROC) graph, the orange line displays the models true positive rate and false positive rate at various thresholds, the dashed blue line represents an AUC of 0.5, no better than chance.

The AUC‐ROC metric refers to the area under the receiver operating characteristic curve, which plots the true positive rate (recall) against the false positive rate at different classification thresholds and measures the entire area under this curve, from (0, 0) to (1, 1) (Figure 1) and is sometimes referred to as a model's predictive capacity [17, 36, 39, 48]. A higher AUC indicates the model is better at distinguishing between positive and negative classes across thresholds and does not require setting a single classification threshold‐like accuracy. Accuracy—amongst others—relies on setting a specific classification threshold, such as 0.5, to map prediction scores to discrete classes. Values above that set threshold are classified as positive, while values below are negative. The AUC value ranges from 0 to 1, with 1 being perfect classification. A drawback is that a high AUC can sometimes overstate model performance if there is a large false positive rate [17, 39]. Still, AUC‐ROC is commonly used in medicine, biometrics and other applications where understanding the trade‐off between true positives and false positives is important [48]. The MCC is another evaluation metric that summarises the confusion matrix into a value between −1 and +1, with higher scores indicating better classification performance. An MCC of +1 represents perfect prediction, 0 is equivalent to random guessing and −1 indicates total disagreement between predictions and true labels.

Compared to metrics like accuracy or AUC‐ROC, MCC provides a more balanced assessment when evaluating imbalanced data sets. Given the relative benefits of the MCC metric, it is recommended to complement the AUC‐ROC score with MCC calculation since it can give a more balanced point of comparison across different model types and data sets [6].

### Human evaluation

Human evaluation is required for generative outputs like text and images. Automatic metrics have limitations, so expert judges should evaluate quality, coherence and correctness, considering the limitations of quantitative evaluation methods. However, human evaluation can be subjective and inconsistent between judges [30]. It is prudent to report interrater agreement measures when using multiple human evaluators of performance [19, 26].

### Offline versus online performance

Offline ML, also called batch learning, describes engineering a model trained on a fixed training set, evaluated by a fixed test data set (internal validation), without changing them during the iteration process. This is the most commonly used type in the medical literature. Concept drift and covariate shifts are not considered after deployment as the model is based on an original data set. Online ML takes into account evolving learning environments and changes in real‐world performance are immediate [36]. In the context of the healthcare sector, both approaches can be utilised with offline learning, for example, being suited for image recognition and classification, while online learning is more suitable for data sets with continuous data streams such as prediction of hospital capacity utilisation. However, even online ML has many risks and the model can drift due to invalid or erroneous data, faulty sensors and so on.

### Uncertainty and interpretability

All models should provide uncertainty estimates like confidence intervals, as point estimates are insufficient in presenting the complete picture, especially in small data sets [1]. Scoring models can also help in this regard since they are both interpretable, for example, it is clear from their construction why they predict a certain outcome and directly predict patient risk via their score [50].

In summary, while metrics provide quantification, responsible evaluation goes deeper to test model limitations and ensure outputs are sound. Evaluation is an iterative process, not a one‐time event. Interdisciplinary collaboration between technical and subject matter experts grounds evaluation in real‐world needs. With rigorous and thoughtful evaluation, we can realise AI's benefits while building trust through transparency. Guidelines and reporting standards have already been proposed for the judicial use of AI and ML models in medical applications and should be consulted both in scientific reporting and in clinical evaluation of AI‐based solutions such as transparent reporting of a multivariable prediction model for individual prognosis or diagnosis (TRIPOD): the TRIPOD Statement [9, 12].

## CASE STUDIES

### Case study 1: Image recognition

Cho et al. analysed 1394 arthroscopic rotator cuff repair images from 580 patients. Images were categorised as 1138 nonretear and 256 retear based on magnetic resonance imaging follow‐up within 2 years postoperatively [7]. The authors implemented standard ML practices for model development and validation. The image data set was split into training (80%) and held‐out test (20%) sets. The training set was further divided into three folds for stratified *k*‐fold cross‐validation to fine‐tune model hyperparameters and prevent overfitting. Performance metrics including accuracy, AUC‐ROC, sensitivity, specificity and F1 − score were reported for both cross‐validation and final model evaluation on the unseen test set. Three pretrained convolutional neural network architectures (VGG16, DenseNet121, Xception) were initialised with transferred weights and fine‐tuned on the training folds. The models achieved cross‐validation accuracy of 80%–99% across folds. On final model testing, DenseNet121 performed best with 91% accuracy, 0.92 AUC, 84% sensitivity and 93% specificity in predicting retearing from the arthroscopic images [7]. Limitations also noted by the authors were the imbalance of the retear to nonretear classes and lack of external validation.

By implementing robust ML methodology including cross‐validation and reporting performance on an unseen hold‐out test set, the authors have demonstrated that deep‐learning analysis of arthroscopic rotator cuff repair images can accurately predict postoperative integrity.

### Case study 2: Database analysis and prediction

Martin et al. analysed data from 62,955 patients in the Norwegian and Danish ACL reconstruction registries [33]. The aim was to develop an ML model to predict risk of revision surgery at 1, 2 and 5 years postoperatively. The data set was randomly split into 75% training and 25% test sets. Four algorithms were tested: Cox lasso regression, random survival forest, gradient boosting machines and super learner ensemble [40]. Hyperparameters were optimised via grid search with cross‐validation on the training set. Performance was evaluated on the test set using Harrell's concordance index (C‐index) for predictive discrimination and Hosmer–Lemeshow calibration plots. Multiple imputation was used to assess potential bias from missing data. The nonparametric ML models (random survival forest, gradient boosting, super learner) demonstrated moderate predictive performance, with indices around 0.67. Despite the large sample size, this was similar to prior models developed using the Norwegian ACL registry alone [32]. A key weakness noted by the authors was that, despite using multiple ML methods, the prediction accuracy for knee revision surgery outcomes showed limited improvement compared to previous simpler models, likely due to substantial missing preoperative data.

By splitting data into training and test sets, tuning hyperparameters via cross‐validation and evaluating discrimination and calibration, the authors implemented rigorous ML methodology. However, model accuracy reached a performance ceiling, indicating that enhancing variable capture may be needed to improve predictions.

## CONCLUSION

In conclusion, as AI continues advancing at a rapid pace, adopting rigorous evaluation, and reporting standards is imperative. The methodologies outlined here, including thoughtful selection of performance metrics, testing on real‐world data distributions, assessing model uncertainties and transparent reporting of details based on existing guidelines, serve as a framework for critical model appraisal. There is still significant work needed to realise AI's potential benefits while mitigating risks. Model interpretability and explainability techniques must continue advancing to enable practitioners to understand how systems reach conclusions. Moving forward, a cross‐disciplinary emphasis on rigorous analytical evaluation, clinical collaboration and ethical deployment will help foster continued AI progress. This will require commitment from researchers, clinicians, journal editors and regulatory agencies alike, to uphold AI evaluation and reporting standards that match these powerful technologies' capabilities and societal impacts.

## AUTHOR CONTRIBUTIONS

All listed authors have contributed substantially to this work: Felix C. Oettl, Ayoosh Pareek, Bálint Zsidai and Eric Hamrin Senorski performed literature review and primary manuscript preparation. Editing and final manuscript preparation were performed by Philipp W. Winkler, Sebastian Kopf, Christophe Ley, Elmar Herbst, Jacob F. Oeding, Alberto Grassi, Michael T. Hirschmann, Volker Musahl, Kristian Samuelsson, Thomas Tischer and Robert Feldt. All authors read and approved the final manuscript.

## CONFLICT OF INTEREST STATEMENT

The authors declare no conflict of interest.

## ETHICS STATEMENT

The authors have nothing to report.
